# Predicting the Extent of Resection of Motor-Eloquent Gliomas Based on TMS-Guided Fiber Tracking

**DOI:** 10.3390/brainsci11111517

**Published:** 2021-11-16

**Authors:** Francesco Belotti, Mehmet Salih Tuncer, Tizian Rosenstock, Meltem Ivren, Peter Vajkoczy, Thomas Picht

**Affiliations:** 1 Neurochirurgische Klinik und Hochschulambulanz Charité—Universitaetsmedizin Berlin, 10117 Berlin, Germany; mehmet.tuncer@charite.de (M.S.T.); tizian.rosenstock@charite.de (T.R.); meltem.ivren@charite.de (M.I.); peter.vajkoczy@charite.de (P.V.); thomas.picht@charite.de (T.P.); 2 Neurosurgery Unit, Spedali Civili di Brescia Hospital, 25123 Brescia, Italy; 3 Berlin Institute of Health (BIH), Anna-Louisa-Karsch-Str. 2, 10178 Berlin, Germany; 4 Cluster of Excellence: “Matters of Activity. Image Space Material”, Humboldt University, 10117 Berlin, Germany

**Keywords:** nTMS, fiber tracking, glioma, extent of resection, outcome

## Abstract

Background: Surgical planning with nTMS-based tractography is proven to increase safety during surgery. A preoperative risk stratification model has been published based on the M1 infiltration, RMT ratio, and tumor to corticospinal tract distance (TTD). The correlation of TTD with corticospinal tract to resection cavity distance (TRD) and outcome is needed to further evaluate the validity of the model. Aim of the study: To use the postop MRI-derived resection cavity to measure how closely the resection cavity approximated the preoperatively calculated corticospinal tract (CST) and how this correlates with the risk model and the outcome. Methods: We included 183 patients who underwent nTMS-based DTI and surgical resection for presumed motor-eloquent gliomas. TTD, TRD, and motor outcome were recorded and tested for correlations. The intraoperative monitoring documentation was available for a subgroup of 48 patients, whose responses were correlated to TTD and TRD. Results: As expected, TTD and TRD showed a good correlation (Spearman’s ρ = 0.67, *p* < 0.001). Both the TTD and the TRD correlated significantly with the motor outcome at three months (Kendall’s Tau-b 0.24 for TTD, 0.31 for TRD, *p* < 0.001). Interestingly, the TTD and TRD correlated only slightly with residual tumor volume, and only after correction for outliers related to termination of resection due to intraoperative monitoring events or the proximity of other eloquent structures (TTD ρ = 0.32, *p* < 0.001; TRD ρ = 0.19, *p* = 0.01). This reflects the fact that intraoperative monitoring (IOM) phenomena do not always correlate with preoperative structural analysis, and that additional factors influence the intraoperative decision to abort resection, such as the adjacency of other vulnerable structures. The TTD was also significantly correlated with variations in motor evoked potential (MEP) responses (no/reversible decrease vs. irreversible decrease; *p* = 0.03). Conclusions: The TTD approximates the TRD well, confirming the best predictive parameter and giving strength to the nTMS-based risk stratification model. Our analysis of TRD supports the use of the nTMS-based TTD measurement to estimate the resection preoperatively, also confirming the 8 mm cutoff. Nevertheless, the TRD proved to have a slightly stronger correlation with the outcome as the surgeon’s experience, anatomofunctional knowledge, and MEP observations influence the expected EOR.

## 1. Introduction

It is widely accepted that the extensive resection of malignant gliomas can improve patient survival and quality of life (QoL) [1,2,3,4]. However, this goal must be balanced with the preservation of neurological functions to maintain the postoperative functional status [5,6,7]. Surgical planning with diffusion tensor imaging tractography (DTI) [8,9] and intraoperative neurophysiological monitoring (IOM) has been proven to increase safety during the resection of rolandic brain tumors [10,11]. Regarding the preoperative functional tools, navigated transcranial magnetic stimulation (nTMS) and nTMS-based tractography [12,13,14] have been extensively validated for the noninvasive analysis of the spatial relationship between brain tumors and eloquent cortical and subcortical structures [15,16,17,18], enabling more extensive resections while reducing the rate of functional deficits [13,18,19,20]. The methods of language nTMS-based tractography are not so well defined, and a risk prediction model has yet to be validated for language. On the contrary, a risk stratification model regarding motor outcome has been published based on infiltration of the primary motor cortex (M1), tract to tumor distance (TTD), and resting motor threshold (RMT) ratio (a parameter of cortical excitability) [21]. It is based on outcome correlation only (not on IOM or postoperative radiological findings), and the most significant variable is TTD [21]. The correlation of TTD with tract to resection cavity distance (TRD) and outcome is a missing link to further evaluate the validity of the risk model (especially the TMS-based tracts).

The aim of this study was to use the early postoperative MRI derived resection cavity, employing state-of-the-art image fusion and distortion correction technology, to measure how closely the resection cavity approximated the preoperatively calculated corticospinal tract (CST) and how the TRD correlates with the TTD, included in the preoperative risk model, and the outcome. This will help us to better understand the relevance of the risk model in general, and the TMS-based tractography TTD specifically.

## 2. Materials and Methods

### 2.1. Ethics

The experimental protocol was approved by the local ethics committee of the Charité University Hospital Berlin in accordance with the Declaration of Helsinki (EA1/016/19). Written and informed consent for all medical evaluation and treatment was obtained from all patients.

### 2.2. Patients and Study Design

A retrospective data collection, including 183 patients who underwent surgical resection for presumed motor-eloquent gliomas from August 2011 to August 2019, was performed. The following inclusion criteria were used: the presence of a glioma, judged according to anatomical magnetic resonance imaging (MRI) to compress or infiltrate the motor cortex and/or be closely related to the CST; and the availability of preoperative nTMS mapping and DTI. All findings were recorded in a custom-made database. The following biographical and clinical items were documented: age, sex, location, histology according to the World Health Organization (WHO) classification [22], motor status according to the British Medical Research Council (MRC) grading (preoperative and postoperative at seven days and three months), and IOM data, available for a subgroup of 48 patients. The functional outcome was based on improved, stable, or worsened motor status (comparing the preoperative MRC status and those on the day of discharge and after three months) and defined by the postoperative development of a persistent new deficit, as previously done by our group [23].

### 2.3. Image Acquisition

Cerebral MRI with a T1 contrast-enhanced 3D gradient echo sequence, fluid-attenuated inversion recovery (FLAIR) sequence, and DTI sequence was performed using a 1.5- or 3-T MRI unit (GE Healthcare; Chicago, IL, USA) with an eight-channel head coil, as previously described in detail [21,24] (see Table S1). An interdisciplinary team of neurosurgeons and neuroradiologists interpreted all MRI scans [21]. The T1 contrast-enhanced 3D gradient echo sequence was also used in the nTMS system (eXimia, Nexstim Oy; Helsinki, Finland) for the mapping of the motor cortex. For the analysis of preoperative images, T1-weighted gadolinium-enhanced (for high-grade tumors), T2-weighted, and FLAIR sequences (for low-grade tumors) were used to calculate the tumor volume using Brainlab Elements software (BRAINLAB AG, Munich, Germany) [21]. Postoperatively, T1- and T2-weighted datasets were used to identify and manually segment the resection cavity. Intra- and postoperative images were carefully analyzed and the distortion correction algorithm, integrated in Brainlab Elements, was used to minimize inaccuracies caused by brain shift and edema [25].

### 2.4. Navigated TMS

nTMS examinations were performed as specified previously [16,21]. In brief, a figure-eight TMS coil, generating a short-lasting, cone-shaped magnetic field, is used to induce an electrical field in the underlying brain. Stimulation of pyramidal cells, their axons, or surrounding interneurons may result in a motor evoked potential (MEP) depending on the stimulation location and intensity [15,26]. MEPs were recorded by the system’s integrated electromyography unit using surface electrodes (abductor pollicis brevis, first dorsal interosseous (FDI), and adductor digiti minimi muscles for the upper extremity; and the tibialis anterior and abductor hallucis brevis muscles for the lower extremity) [27]. The RMT, defined as the lowest stimulation intensity sufficient to induce a MEP (≥50 μV) in at least five of 10 stimulations, based on the “hotspot” [27] of the FDI muscle, was used for the peritumoral mapping of the upper (stimulation intensity: 110% RMT) and lower (median stimulation intensity: 130% RMT) extremities [21]. Finally, mapping with high specificity (stimulation intensity: 105% RMT) was performed to specifically outline the primary motor cortex along the precentral gyrus, as previously described [21].

### 2.5. Tractography

The TMS stimuli locations outlining the primary motor cortex were imported into Brainlab iPlan 2.0 surgical planning software in the DICOM format (BRAINLAB AG, Munich, Germany). The fiber tracking reconstruction tool employed in our study is provided by Brainlab, as part of the commercial neuronavigation software, and it is based on FACT and TEND deterministic algorithms. The calibration of this commercial tool was previously described [28]. The nTMS-positive spots were enlarged to a radius of 3 mm to generate a continuous seed point area [24]. To improve tracking robustness, a second seed point was placed in the anterolateral portion of the ipsilateral cerebral peduncle [12]. Afterward, fiber tracking at 75% of the fractional anisotropy threshold, with a minimum fiber length of 110 mm, was performed, as described in detail elsewhere [21,24]. Clearly aberrant tracts were removed, and the minimum distance between the tumor and the CST was measured, defining the TTD [29].

### 2.6. IOM

The IOM documentation was available for a subgroup of 48 patients. A standardized procedure consisting of monopolar anodal trains of five square-wave pulses (0.3 ms, 400 Hz) for cortical and subcortical mapping, as well as monitoring of motor function, was applied, as previously described [11,13]. Data regarding intraoperative transient or persistent decrease in MEPs and stimulation intensity values were recorded.

### 2.7. Postoperative Image Analysis

**Figure 1:**
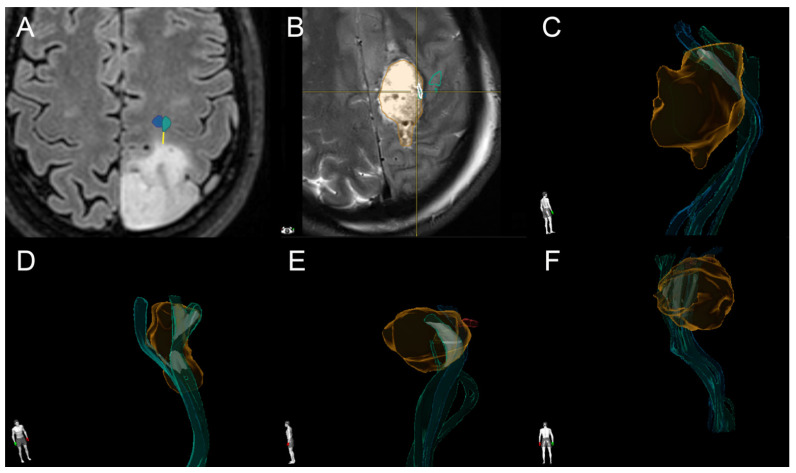
TTD and TRD measurement. (**A**) An example of TTD measurement (yellow line) from the hand portion of the CST (green object) in one patient from our sample; (**B**) an example of intersection (TRD = 0) of the resection cavity (orange volume) with the CST (green and light blue objects) in another patient; (**C**) a 3D reconstruction of the volume of the resection cavity and the CST in the same patient as in (**B**) (the white volume corresponds to the intersection); (**D**–**F**) 3D reconstructions of the volume of the resection cavity and the CST in three other patients from our sample.

The postoperative images were fused to the preoperative MRIs and nTMS-based tractographies using Brainlab Elements software. The distortion correction tool included in the software, based on semielastic image fusion, was applied to minimize the misalignments caused by brainshift [25,30]: firstly, the preoperative nTMS-based CST was fused to the T1-weighted sequence; then the preoperative and postoperative MRI sequences were fused together, deforming the preoperative landmarks according to their position on the postoperative MRI; lastly, the preoperative CST was deviated according to the postoperative morphology of the brain. The distortion correction and the tractography reconstruction were done automatically, using the corresponding modules of the Brainlab neuronavigation system: this commercially available software cannot be modified for research purposes and the results were not compared to other software analyses. The relationships of the CST to the resection cavity were evaluated independently. In case of the overlap of a tract and the resection cavity (=tract injury), the volume of the intersection was calculated using the object manipulation tool [30]. Otherwise, the distance of the resection cavity from the CST was measured (Figure 1), defining the TRD [30].

### 2.8. Statistics

SPSS Statistics software (IBM, Armonk, NY, USA) was used for the statistical analysis. Descriptive analysis was performed for each variable to describe the sample (mean, SD, range for demographic data; mean, SD, median, interquartile range for volumes). The correlation between TTD, TRD, and residual volume (RV) was analyzed using Spearman’s rank correlation coefficient (ρ). The correlation analysis was also repeated after grouping patients according to TTD and TRD ordinal categories as follows: intersection, 0–8 mm distance, >8 mm distance [21]. Considering that both variables had only three categories, we investigated the correlation using Kendall’s Tau-b test.

The same TTD and TRD groups and Kendall’s Tau-b test were used to investigate the correlation between the distances and the motor outcome at three months, defined as the development of a persistent new deficit. Receiver operating characteristic (ROC) curves, with the analysis of the area under the curve (AUC), were also used to assess the specificity and the sensibility of TTD and TRD (individually but also in conjunction, using a logistic regression) to predict the motor status at seven days and at three months.

The analysis of the subgroup of patients that underwent IOM during the surgical operation was based on MEP responses, classified as absent or reversible decrease and persistent decrease, and TTD and TRD measurements using the Mann–Whitney U test.

## 3. Results

### 3.1. Characteristics of Patients and Tumors

Sixty-nine women and 114 men, with a mean age of 50 years, were included. The male to female ratio was approximately 1.5:1 (M: 62%, F: 38%). Tumors were most frequently located within the frontal lobe. A detailed overview of patients’ characteristics is presented in Table 1.

### 3.2. Analysis of TTD and TRD

**Figure 2:**
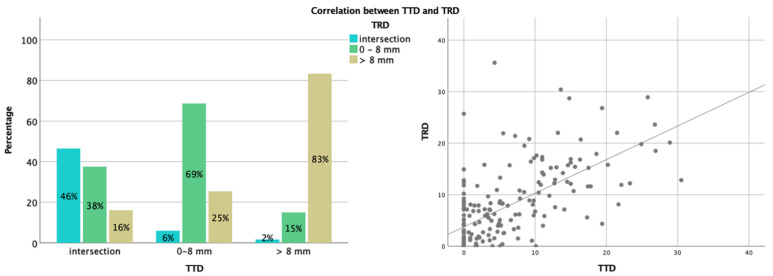
Correlation between the TTD and the TRD. The figure shows a bar chart for TRD according to TTD categories (8 mm cutoff and intersection) on the left side. On the right side, the scatter plot shows the correlation between TTD and TRD as continuous variables.

There was a positive correlation between TTD and TRD (Spearman’s ρ = 0.67, *p* < 0.001). Figure 2 illustrates this association according to categorized values of TRD and TTD (Kendall’s Tau-b coefficient = 0.58, *p* < 0.001).

### 3.3. Correlation between TTD, TRD, and Extent of Resection (or Residual Volume)

There was a weak positive correlation between the TTD and extent of resection (EOR) (Spearman’s ρ = 0.32, *p* < 0.001), and a negative correlation with RV (ρ = −0.32, *p* < 0.001), showing that the lower the TTD, the higher the probability of residual volume. In detail, we observed that when TTD was ≥8 mm, GTR was obtained in 85.0% of cases, in 65.7% of cases when TTD was 0–8 mm, and in only 43.64% of cases when the tumor intersected the CST. Almost no correlation was seen between TRD and EOR (ρ = 0.02, *p* = 0.78) or RV (ρ = −0.02, *p* = 0.83).

### 3.4. Functional Outcome

The majority of patients (178, 97.3%) had a motor status of MRC grade 3 or higher before surgery. Postoperatively, 44 patients (24.0%) developed a new deficit or worsened preexisting hyposthenia (lower grade of MRC). After three months, 30 (18.3%) patients had new motor symptoms (lower grade of MRC compared to before surgery); in 26 (15.9%) of these cases, the deficit was persistent compared to the immediate postoperative status. Fifteen (9.2%) cases showed partial recovery.

**Figure 3:**
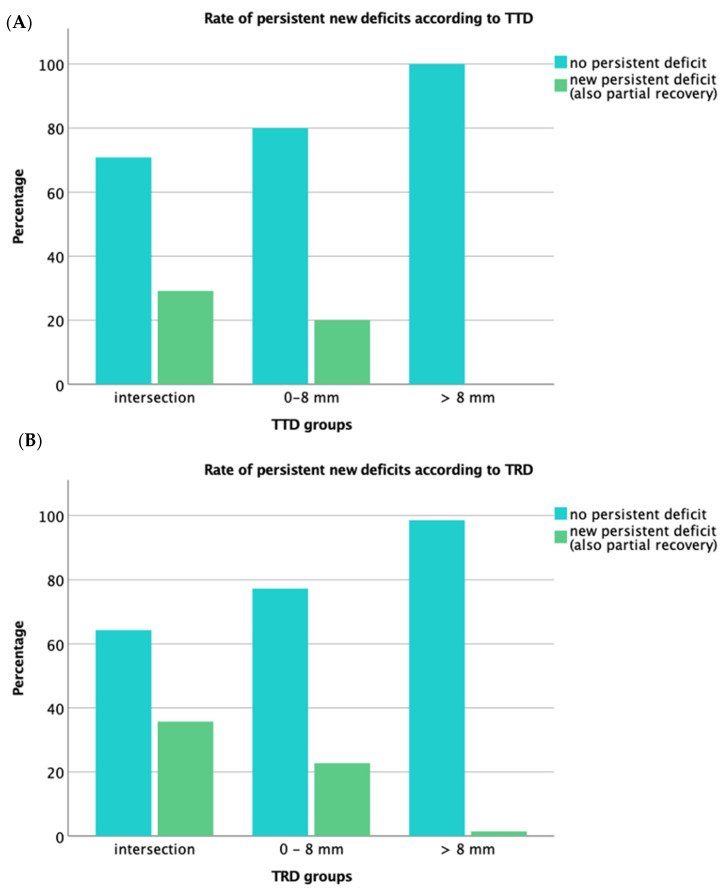
Bar chart for onset of new deficits among the TTD and TRD groups. The figure shows the development of persistent new deficits (defined as “worsened”) at three months among the different categories of TTD (**A**) and TRD (**B**), compared to patients with stable or improved deficits. The TRD seems to better discriminate the outcome according to CST infiltration.

The number of preoperatively infiltrated tracts, and of tracts injured after surgery for unchanged and worsened patients, are presented in Table 2, together with the results of the analysis with Kendall’s Tau-b test, which demonstrated significant differences between the TTD and TRD groups (Figure 3).

The table shows the group comparison of functional outcome after three months, defined as the development of a persistent permanent deficit. The 8 mm cutoff and the tract infiltration, for TTD, and injury, for TRD, were used as cutoffs between the categories. The results of the statistical analysis are also reported. * Note that this patient suffered from postoperative ischemia.

The results of the analysis of the ROC curves for new deficits at 7 days or 3 months in relation to TTD and TRD are reported in Table 3. The TRD performed best in terms of AUC, although the TTD and TTD + TRD curves did not differ significantly.

The table shows AUCs (with 95% confidence intervals) regarding the ROC curves of outcome prediction, according to TTD and TRD values, after seven days and three months. No statistically significant difference was found between the TTD and TRD areas. The curve that originated from the logistic regression (TTD + TRD) did not differ significantly either.

### 3.5. Analysis of the IOM Subgroup

**Figure 4:**
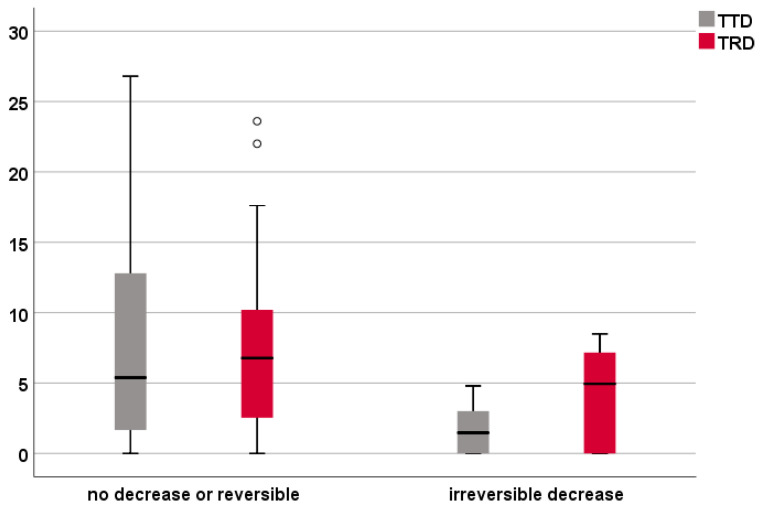
Box plot for the IOM results. The figure shows the TRD and TTD measurements for the two categories of MEP responses, namely patients with no or reversible decrease and patients with an irreversible decrease.

The results of the statistical analysis regarding the IOM subgroup are presented in Figure 4 and Table 4. The Mann–Whitney U test was used to investigate the correlation of TTD and TRD with the results of IOM. Only the TTD showed a significant correlation with MEP responses (*p* = 0.033).

The table shows the results of the Mann–Whitney U test that was used to investigate the correlation between the MEP variations and the different categories of TTD and TRD.

## 4. Discussion

The main goal of brain tumor surgery is to achieve an extensive but functionally safe resection of the tumor [5]. This concept is essential for tumors located in eloquent areas, where the risk of inducing a new functional deficit is concrete. The postoperative worsening of the functional status decreases the patient’s QoL, sometimes hampering access to adjuvant treatments, and correlates with shorter survival [7]. Previous works proved that preoperative nTMS mapping in patients with a brain tumor in a presumed motor-eloquent location reduced the rate of permanent motor deficits [13,20,21]. Furthermore, nTMS-based tractography has been demonstrated to increase the accuracy of fiber tracking in a user-independent manner compared to conventional fiber tracking [18,21,24]. The clinical utility of nTMS has been further improved via a recently described model of nTMS-based risk stratification, allowing us to objectively identify cases at high risk of incurring a new postoperative motor deficit, therefore facilitating preoperative risk–benefit balancing and patient counseling [21]. One of the variables included in the model was TTD with a safety distance of more than 8 mm, which, without other risk factors, defines a low risk of new deficits [21].

In our study, the correlation between TTD and TRD, which is obtained postoperatively as the distance between the resection cavity and the brainshift-corrected nTMS-based CST, was tested. TTD and TRD showed a good correlation, both as continuous variables and as categories with the 8 mm cutoff. Therefore, if the preoperative planning is adequate and followed during surgery, the TTD included in the risk stratification model satisfactorily estimates the MEP-controlled distance between the CST and the resection cavity [21].

The correlation analysis between the TTD and the RV showed a negative relationship, confirming the role of nTMS-based DTI in preoperative surgical planning to preserve the functional status of the patient; the same results were confirmed by the positive correlation between the TTD and the EOR. Nevertheless, the weakness of the correlation between the TRD and the RV could reflect the role of other factors to determine the extent of resection, such as the proximity of the tumor to inviolable anatomical sites other than the motor system (31.4% of cases in our study), IOM responses during surgery (51.4% of cases in our study), and the possible premature termination of the resection (17.2% of cases in our study) [31]. Furthermore, we identified 13 outlier cases influencing the slope of the regression lines from the comparison between the scatterplots correlating the EOR with the TTD and TRD, respectively (i.e., cases with significant difference between TRD and TTD). The two measures differed widely due to MEP decreases, and consequent resection stopped, in five patients (2.7%), due to the removal of the high-grade portion only in three (1.6%), and due to surgical planning in five cases (2.7%) presenting infiltration of the callosal body, insula, or planned for subtotal resection or open biopsy. After removing these cases, the Spearman’s correlation between EOR and TTD remained significant, and the correlations between the TRD and the EOR (ρ = 0.19, *p* = 0.01) or the RV (ρ = −0.19, *p* = 0.01) also became significant. Among the five resections stopped based on IOM responses, two cases had infiltration of M1 (area outside the conservative DTI algorithm employed), one case of insular glioma had a MEP decrease during the resection along its medial surface, one exeresis was stopped when MEP was evoked at 10 mA (estimate distance higher than TTD), and in the last case the extensive infiltration of the CST led to a partial resection after an intraoperative MRI documenting a TRD of 6 mm. Taking into account the slight correlation of the TRD with the GTR that emerges (excluding outliers), we identified four possible reasons behind the discrepancy between the TTD and the TRD:

1. Resection stopped according to IOM to avoid postoperative deficits, leading to an EOR lower than planned (unexpected MEP signal);
2. MEP decrease at a certain distance from the CST, as planned, but reaching that distance in only one trajectory (expected MEP response as stop signal, but overestimating the surrounding resection);
3. Resection stopped for other anatomical reasons;
4. Premature termination of resection due to unclear reasons.

The data on the functional outcome confirmed that patients with direct tumor infiltration of the CST had the highest rate of motor worsening. Both the TTD and the TRD showed a significant correlation with the onset of persistent new deficits at three months, although the TRD one was stronger. The same results were confirmed by the analysis of the ROC curves based on TTD and TRD to estimate the outcome after seven days and three months. A slightly higher AUC was obtained with TRD values, although the *p*-values of the differences with the TTD were not significant. Even taking into account both measurements, the ROC curve did not differ significantly. The slightly better performance of the TRD could be due to a better distinction between stable or improved deficits and permanent deficits, especially in the case of CST injury, as shown in Figure 3. The data were confirmed by the slightly higher percentage of persistent new deficit when the CST intersected with the tumor based on the TRD (10 cases, 35.7%) compared to the TTD (14 cases, 29.2%). On the other hand, based on these results, resections were performed within the course of the CST without new persistent deficits in 64.3% of cases. Although this could be due to the inability of the distortion correction algorithm to compensate for brainshift or inaccuracy of the preoperative tractography, the fact that most of these cases already had the intersection preoperatively, as well as the correlation between TTD and TRD, may support the notion of a compensating mechanism of motor function, especially since a conservative DTI algorithm and FA thresholding [24], somewhat underestimating the real extent of the CST, were used. Our results on functional outcome also upheld the 8 mm cutoff previously identified [21]. Indeed, none of the patients with TTD and TRD > 8 mm developed postoperative deficits (except in one case, related to an ischemic event). The lower rate of TRD intersections, compared to the TTD, suggests the role that IOM plays during surgery, and may be an additional factor leading to the slightly stronger correlation of the TRD with the outcome.

Regarding the data on IOM, we recorded only six patients with an irreversible decrease in MEPs, probably thanks to presurgical planning and the availability of nTMS-based DTI, possibly introducing selection bias. The TTD was significantly correlated to variations in MEP responses; an irreversible decrease was seen only during surgery for tumors closer than 3.5 mm to the CST. One of these six patients had a TTD of 0 mm and a TRD of 8.5 mm, making it difficult to confirm statistical significance for the TRD.

Nevertheless, the TRD values were globally higher than the TTD ones (23 patients versus 19 in the 0–8 mm group, and seven patients versus 12 in the intersection group). In our opinion, this is due to the interruption of resection along the tumor border where the MEPs decreased; therefore, a small residual could have been left to avoid further impairment of the CST. These results may suggest a potentially higher reliability of the TRD, thanks to the additional effect of IOM, leading to a better functional outcome.

Based on our findings, TTD was confirmed to play a key role in predicting the RV and the IOM responses. TRD, instead, is slightly more reliable to estimate the motor outcome at three months in terms of the onset of new persistent deficits. However, the TTD and the TRD are strongly correlated, and the TTD has highly comparable specificity and sensibility, based on AUCs. Therefore, the predictive model based on TTD could be one of the options available for surgical planning and patient consultation.

Together, our results on EOR, IOM, and functional outcome, also taking into account the possible reasons for discrepancies between the TTD and the TRD, suggest that, given the high accuracy of the neuronavigation, sticking to preoperative planning leads to the best results in terms of EOR. IOM allows for following the surgical strategy intraoperatively, and also achieving the best functional outcome. The evidence that, in some cases, some residual tumor is found postoperatively, despite the functional border being reached during surgery, may be an indication of the need for intraoperative MRI or ultrasonography, if available. Such technologies are not so widespread, due to costs and low accessibility. Nevertheless, intraoperative imaging is theoretically the only method adding radiological information to the functional data of IOM. Awake mapping is one of the mainstays of glioma surgery in eloquent areas; it is not discussed here because it is beyond the scope of this article.

### Limitations

The analyses in our study are solely dependent on the radiographic results, correlated with postoperative motor status. Despite efforts to avoid misalignments during fusion using the distortion correction tool, errors due to brainshift or variable scanning procedures cannot be ruled out, as pointed out previously by our group [30]. Tractography itself suffers from a variety of limitations that render its routine use difficult [30,31]. It is known that tractography results contain false positive and false negative streamlines [32,33]. Moreover, tractography cannot recognize the difference between afferent and efferent connections, and streamlines may terminate improperly [34]. DTI-based tractography can be used to reconstruct major white matter pathways but only describes the average fiber orientation per voxel and is not capable of resolving the problem of crossing fibers [31]. Moreover, the subjectivity of RV measurements and the approximation due to the DTI and distortion correction algorithms may have affected the estimation of EOR and TRD; besides, the 8 mm cutoff has not been directly determined in our study, but it was set according to Rosenstock et al. [21]. Nevertheless, our results confirmed the safety of that distance for motor outcome.

In this study, we have focused on the analysis of the white matter and correlated its injury to the functional outcome. Deficits that may have occurred due to cortical lesions only or because combined cortical and subcortical lesions were not detected separately, but a partial correlation analysis, correcting for motor cortex infiltration, was performed and confirmed the results [30].

Finally, the analysis regarding the IOM may have suffered from selection bias due to the small number of patients with an irreversible decrease in MEPs.

## 5. Conclusions

The TRD provides a reliable estimate of persistent postoperative motor deficits with acceptable AUC predictive values. The TTD was confirmed to be a very reliable predictor for RV and EOR. Our results show that TTD predicts TRD well, which strengthens the nTMS-based risk stratification model [21]. However, the TRD had a stronger correlation with motor outcome at three months than the TTD. This can be explained by independent factors that influence the projected EOR such as (unexpected) intraoperative IOM phenomena, the individual surgeon’s experience, and implicit anatomofunctional knowledge.

## Figures and Tables

**Table 1:** Patients’ characteristics. * One patient had a multicentric tumor located in the right frontal lobe and in the left parieto-occipital lobes. The term *insular tumors* include also those extending to the frontal, temporal, and parietal lobes.

**Demographic Characteristics**						
Age—years		mean (SD) [range]			50 (16) [21–81]	
Sex—no. (%)		males 114 (62%)			females 69 (38%)	
**Clinical characteristics**						
**Preoperative motor function (MRC) no. (%)**						
0		4 (2.2%)				
1		2 (1.1%)				
2		--				
3		14 (7.7%)				
4		38 (20.7%)				
5		126 (68.9%)				
		**Functional outcome at 7 days after surgery—no. (%)**			**Functional outcome at 3 months after surgery—no. (%)**	
Improved		18 (9.8%)			22 (13.4%)	
Stable		121 (66.1%)			111 (67.7%)	
Worsened		44 (24.0%)			31 (18.9%)	
**Tumor characteristics**						
**Tumor Location—no. (%)**						
**Frontal**		**Parietal**		**Temporal**		**Insular**
total	68 * (37.2%)	total	52 (28.4%)	total	15 (8.2%)	23 (12.6%)
precentral	17 (9.3%)	postcentral	23 (12.6%)	temporo-occipital	1 (0.5%)	
fronto-temporal	4 (2.2%)	parieto-occipital	6 * (3.3%)			
fronto-parietal (central region)	27 (14.8%)	parieto-temporal	2 (1.1%)			
		parieto-temporo-occipital	1 (0.5%)			
**WHO tumor grade—no. (%)**			**Affected hemisphere—no. (%)**			
I		3 (1.6%)	right		98 (53.6%)	
II		29 (15.8%)	left		84 (45.9%)	
III		44 (24.0%)	both		1 (0.5%)	
IV		107 (58.5%)				
**Tumor volume and surgical results**						
		Mean (SD)			Median (interquartile range—IQR)	
Tumor volume—mL		39 (38)			26 (13–53)	
Residual volume—mL		5 (15)			0 (0–2)	
Extent of resection—%		91.9 (18)			100 (95–100)	

**Table 2:** Preoperative infiltration of tracts, tract injury, and outcome.

CST Infiltration	*n*	Persistent New Deficit (*n*=): Infiltration (%)	*p*-Value	Kendall’s Tau-b
TTD				
intersection	48	14 (29.2%)	<0.001	0.30
0–8 mm	60	12 (20.0%)		
>8 mm	56	0 (0%)		
TRD				
intersection	28	10 (35.7%)	<0.001	0.35
0–8 mm	66	15 (22.7%)		
>8 mm	70	1 (1.4%) *		

**Table 3:** AUC for new deficits in relation to TTD and TRD.

	New Deficit at 7 Days (*n* = 183)	New Deficit at 3 Months (*n* = 164)
TTD	0.74 (0.67–0.82)	0.66 (0.57–0.76)
TRD	0.79 (0.72–0.86)	0.72 (0.63–0.81)
*p*-value for comparison of ROC curves	0.241	0.137
TTD + TRD	0.80 (0.67–0.82)	0.72 (0.63–0.81)

**Table 4:** Correlation of TTD and TRD with the IOM results.

		Cases with No Decrease or Reversible (*n* = 42)	Cases with Irreversible Decrease (*n* = 6)	P (Mann–Whitney U Test)
TTD	intersection	9	3	
	0–8 mm	16	3	
	>8 mm	17	0	
	median (IQR)	5.4 (1.6–12.8) mm	1.5 (0–3.5) mm	0.033
TRD	intersection	5	2	
	0–8 mm	20	3	
	>8 mm	17	1	
	median (IQR)	6.8 (1.4–10.6) mm	4.9 (0–7.5) mm	0.249

## Data Availability

The data presented in this study are available on request from the corresponding author. The data are not publicly available due to privacy restrictions.

## Supplementary Materials

The following are available online at https://www.mdpi.com/article/10.3390/brainsci11111517/s1, Table S1: Magnetic resonance imaging (MRI) acquisition parameters.
