# Supplementary material for: Predicting the Extent of Resection of Motor-Eloquent Gliomas Based on TMS-Guided Fiber Tracking
Source: Brain Sci. 2021 Nov 16;11(11):1517. doi: 10.3390/brainsci11111517 (PMC8615964; doi:10.3390/brainsci11111517)
Supplement: Supplementary file 1 [file brainsci-11-01517-s001.zip › brainsci-1424797-supplementary.pdf]

**Preoperative and postoperative MRI**	
T1-weighted fast spin-echo sequence	TR/TE 565 ms/14 ms
T2-weighted fast spin-echo sequence	TR/TE 5200 ms/100 ms
T2-weighted inversion recovery fast spin-echo sequence	TR/TE/TI 6000 ms/ 150 ms/ 2000 ms
T2*-weighted gradient-echo sequence	TR/TE/α 800 ms/30 ms/20°
1.0-mm section thickness T1 inversion recovery 3D gradient-echo sequence (IR 3D-FSPGR)	TR/TE/TI/α 7.8 ms/3.1 ms/500 ms/16°
**DTI sequences**	
Repeated diffusion weighted single-shot echo-planar sequence along 23 different geometric directions at a b-value of 1000 s/mm^2; additional measurement without diffusion weighting (b = 0 s/mm^2)<br>42 contiguous 3 mm thick axial sections (1008 images in total)	TR/TE 11,000 ms/83 ms; matrix size 128 × 128; FOV 240 × 240 mm

Supplemental Table S1. Magnetic resonance imaging (MRI) acquisition parameters. DTI, Diffusion tensor imaging.
